# Editorial: Immunobiology of Osteoarticular Diseases

**DOI:** 10.3389/fimmu.2021.698992

**Published:** 2021-05-13

**Authors:** Erminia Mariani, Daniela Frasca

**Affiliations:** ^1^ Laboratory of Immunorheumatology and Tissue Regeneration, Istituto di Ricovero e Cura a Carattere Scientifico (IRCCS) IstitutoOrtopedico Rizzoli, Bologna, Italy; ^2^ Department of Medical and Surgical Sciences, Alma Mater Studiorum – University of Bologna, Bologna, Italy; ^3^ Department of Microbiology and Immunology, University of Miami Miller School of Medicine, Miami, FL, United States; ^4^ Sylvester Comprehensive Cancer Center, University of Miami Miller School of Medicine, Miami, FL, United States

**Keywords:** rheumatic diseases, osteoarticular diseases, osteoarthritis, rheumatoid anhritis, osteoporosis, Inflammation, immunological mechanisms, diagnosis

Rheumatic and osteoarticular diseases, that include Osteoarthritis (OA), Rheumatoid Arthritis (RA) and Osteoporosis, represent the most diffuse chronic conditions in the elderly. These diseases affect the individual’s working abilities and autonomy, as well as life expectancy. These chronic inflammatory diseases share similar patho-physiological pathways including increased bone remodeling/resorption, a senescence-associated phenotype, and accumulation of activated immune cells and soluble factors in the joints and skeletal tissue.

Despite the awareness of the importance of inflammatory dysregulation, the increasing knowledge that joint tissues contribute to damage in various osteoarticular diseases and that the disease progression can develop over a long period of time, many questions remain unanswered. Moreover, it is not surprising that treatments, either pharmacological or surgical, only partially address the clinical issue. Therefore, a more in-depth understanding of the mechanisms underlying the development of these disorders could lead to earlier intervention and to the identification of alternative, more appropriate, and less invasive therapeutic approaches.

This Research Topic includes 12 contributions from a number of prominent scientists in the field and addresses specific aspects in the field of Immunobiology of Osteoarticular Diseases. Here is a summary of the single contributions.

Paladini et al. review the evolution of the aminopeptidases Endoplasmic Reticulum Aminopeptidases 1 and 2, (ERAP1 and 2) and LNPEP (Leucyl and Cystinyl Aminopeptidase) in the zoological scale, their functions and associations with immune-mediated diseases. These peptidases play a role in the control of the vascular inflammatory response. Later on during evolution they acquired a role in antigen presentation, diversifying between those residing in the ER, (ERAP1 and 2), whose role is to refine the MHC-I peptidomes, and LNPEP, mostly present in the endosomal vesicles contributing to antigen cross-presentation or in the cell membrane as receptor for angiotensin IV. Therefore, their role in autoinflammatory/autoimmune diseases can be as “contributors” to the shaping of the immune-peptidomes as well as “regulators” of the vascular response.

Xu et al. summarize possible roles of PKM2, an isoform of Pyruvate Kinase in the pathogenesis of RA. PKM2, which is increased in the synovial tissue of RA patients, likely regulates the metabolic requirements of activated immune cells and sinoviocytes, needed to infiltrate the tissue, secrete inflammatory mediators and regulate local inflammation. These results have suggested the possibility to block metabolic pathways to reduce synovial inflammation in RA patients.

Rolph and Das highlight the emerging role of KLF (Krüppel-like factor, a family of DNA-binding zinc finger proteins)-2 in regulation of osteoclastic differentiation. KLF2 overexpression inhibits intracellular pathways that depend on IL-1β signaling. More specifically in the context of RA, global deletion of KLF2 attenuates expression of MMP9 and inflammatory cytokines, contributing to elevated osteoclastogenesis and more aggressive disease progression. Overall data demonstrate that this factor may play a protective role in bones and surrounding tissue by attenuating inflammation in arthritic joints.

Möller et al. review how infectious agents present in the mouth or in the gut of RA patients, may have unique citrullinating capacity, and may also be involved in the establishment of local and systemic inflammation. The data presented in the review strongly indicate the potential role of different microbial taxa in the development of the disease.

Dong et al. demonstrate the pathological role of global and specific cell-free (cf) DNA molecules in RA and OA patients. These molecules are released in large amounts in body fluids of patients, more in RA as compared to OA patients, and induce apoptosis, pyroptosis and necrosis. These molecules in RA patients are highly inflammatory, induce the secretion of pro-inflammatory cytokines and are pathogenic for RA patients, suggesting that they can be potential targets of therapeutic intervention.

Nerviani et al. show that the histological analysis of the synovial tissue of RA patients may help to identify patients with lower probability of response to TNF-α blockade. Interestingly, the high baseline presence of inflammatory cell types predict the (better) response to TNF-α blockade.

Miao et al. show that CD147, a T cell activation marker, negatively regulates Th17 responses in RA patients, as well as in a mouse model of collagen-induces arthritis, by limiting their exuberant proliferation, AKT/mTORC1/STAT3 signaling as well as their interaction with inflammatory monocytes. These results have suggested the possible use of anti-CD147 antibodies as therapeutic options for RA patients.

Jiang et al. investigate the function of miR-146a, and its association with levels of regenerating islet-derived protein 3-alpha (REG3A), in regulating macrophage migration in Polymyositis and Dermatomyositis (PM/DM) patients. PM/DM, classified as typical idiopathic inflammatory myopathies, affect mainly proximal muscles and present some overlap with other disorders such as rheumatoid arthritis, supported by the persistent monocytes/macrophages markers in these disease processes. Reduced miR-146a expression in these myopathies leads to increased REG3A expression that increases inflammatory macrophage migration, which may be a possible underlying mechanism of DM/PM pathogenesis.

Assirelli et al. demonstrate that the different joint tissues (cartilage, synovium and bone) release the complements factors C3, C4 and CFB, and IL-1β pro-inflammatory stimulus enhance CFB. Cartilage and synovium tissues, as well as isolated chondrocytes and synoviocytes, were all able to spontaneously secrete complement activating factors (C3a, C5a, CFBa). Conversely, C5b-9 complex release was only detectable in cartilage and synovium tissue supernatants. An association between some C alternative pathway component and joint inflammation is suggested.

Warner et al. has evaluated the role of IL-15, and of genetic variants of the IL-15R gene, in the pathogenesis of cartilage degeneration in OA patients. Results have shown an association between pain and genetic variants of the IL-15R gene, suggesting that IL-15 signaling may be a target for pain. No associations however were found with cartilage matrix loss and radiographic severity.

Lorenzin et al. presented the results of the Italian arm of the SPACE study on Spondyloarthritis (on-going observational prospective cohort multi-center study II level evidence). Spondyloarthritis, a group of chronic inflammatory rheumatic diseases, can be divided into axial (axSpA) and peripheral forms. The first, mainly affecting the spine and sacroiliac joints and beginning at young age, can be further divided between non-radiographic and radiographic axSpA (also known as ankylosing spondylitis). At baseline, a significant prevalence of bone marrow edema lesions was observed both in sacroiliac joints and spine, with predominant involvement of thoracic district. Since positive MRI-spine images were observed in the absence of sacroiliitis, these findings seem to be relevant in the axSpA diagnosis. Early age of disease onset, long duration of low back pain, increased inflammatory biomarkers, higher use of NSAIDs, male gender, HLA-B27 positivity, Spondyloarthritis Research Consortium of Canada (SPARCC) sacroiliac joints score>2 appeared predictors of radiological damage and activity.

Toni et al. highlight the micro-topography of immune cells in the cancellous and cortical bone compartments in relation to the most consistent data on their action in bone remodeling, to offer an innovative space-related perspective of the action of immune cells involved in the osteoporotic process, and to understand how different osteoporotic lesions develop, thus prompting the design of experimental tools for *in vitro* modeling of early phases of the osteoporotic process and related innovative treatments.

## Author Contributions

All authors contributed to the article and approved the submitted version.

## Conflict of Interest

The authors declare that the research was conducted in the absence of any commercial or financial relationships that could be construed as a potential conflict of interest.

